# Development of national consensus statements on food labelling interpretation and protein allocation in a low phenylalanine diet for PKU

**DOI:** 10.1186/s13023-018-0950-z

**Published:** 2019-01-03

**Authors:** British Inherited Metabolic Diseases Group (BIMDG) Dietitians Group, Sharon Evans, Suzanne Ford, Sarah Adam, Sandra Adams, Jane Ash, Catherine Ashmore, Gillian Caine, Rachel Carruthers, Sarah Cawtherley, Satnam Chahal, Anne Clark, Barbara Cochrane, Anne Daly, Karen Dines, Marjorie Dixon, Carolyn Dunlop, Charlotte Ellerton, Moira French, Lisa Gaff, Cerys Gingell, Diane Green, Joanna Gribben, Anne Grimsley, Paula Hallam, Una Hendroff, Melanie Hill, Rachel Hoban, Sarah Howe, Inderdip Hunjan, Kit Kaalund, Eimear Kelleher, Farzana Khan, Steve Kitchen, Karen Lang, Sharan Lowry, Jo Males, Georgina Martin, Nicola McStravick, Avril Micciche, Camille Newby, Claire Nicol, Rachel Pereira, Louise Robertson, Kathleen Ross, Emma Simpson, Kath Singleton, Rachel Skeath, Jacqueline Stafford, Allyson Terry, Ruth Thom, Alison Tooke, Karen vanWyk, Fiona White, Lucy White, Anita MacDonald

**Affiliations:** 1 Dietetic Department, Birmingham Women’s & Children’s NHS Foundation Trust, Birmingham Children’s Hospital, Steelhouse Lane, Birmingham, B4 6NH UK; 2 The National Society for Phenylketonuria, London, UK; 3 Queen Elizabeth University Hospital, Glasgow, UK; 4 Royal Victoria Infirmary, Newcastle upon Tyne, UK; 5 University Hospital of Wales, Cardiff, UK; 6 Mid Yorkshire Hospitals NHS Trust, Yorkshire, UK; 7 University College London Hospitals NHS Foundation Trust, London, UK; 8 Great Ormond Street Hospital for Children NHS Foundation Trust, London, UK; 9 Children’s University Hospital Dublin, Dublin, Republic of Ireland; 10 Belfast Health & Social Care Trust, Belfast, UK; 11 Royal Hospital for Sick Children Edinburgh, Edinburgh, UK; 12 Leicester Royal Infirmary, Leicester, UK; 13 Cambridge University Hospitals NHS Foundation Trust, Cambridge, UK; 14 Nottingham University Hospitals NHS Trust, Nottingham, UK; 15 Salford Royal NHS Foundation Trust, Salford, UK; 16 Evelina London Children’s Healthcare, London, UK; 17 Mater Misericordiae University Hospital Dublin, Dublin, Ireland; 18 Sheffield Teaching Hospitals NHS Foundation Trust, Sheffield, UK; 19 University Hospitals Birmingham NHS Foundation Trust, Birmingham, UK; 20 Bradford Teaching Hospitals NHS Foundation Trust, Bradford, UK; 21 Ninewells Hospital Dundee, Dundee, Scotland; 22 Sheffield Children’s NHS Foundation Trust, Sheffield, UK; 23 Aneurin Bevan University Health Board Wales, Newport, UK; 24 Bristol Royal Hospital for Children, Bristol, UK; 25 Norfolk and Norwich University Hospital, Norfolk, UK; 26 Royal Aberdeen Children’s Hospital, Aberdeen, UK; 27 Royal Manchester Children’s Hospital, Manchester, UK; 28 Alder Hey Children’s NHS Foundation Trust, Liverpool, UK

**Keywords:** Phenylketonuria (PKU), Delphi method, Food labelling, Consensus, Phenylalanine exchanges

## Abstract

**Background:**

In the treatment of phenylketonuria (PKU), there was disparity between UK dietitians regarding interpretation of how different foods should be allocated in a low phenylalanine diet (allowed without measurement, not allowed, or allowed as part of phenylalanine exchanges). This led to variable advice being given to patients.

**Methodology:**

In 2015, British Inherited Metabolic Disease Group (BIMDG) dietitians (*n* = 70) were sent a multiple-choice questionnaire on the interpretation of protein from food-labels and the allocation of different foods. Based on majority responses, 16 statements were developed. Over 18-months, using Delphi methodology, these statements were systematically reviewed and refined with a facilitator recording discussion until a clear majority was attained for each statement. In Phase 2 and 3 a further 7 statements were added.

**Results:**

The statements incorporated controversial dietary topics including: a practical ‘scale’ for guiding calculation of protein from food-labels; a general definition for exchange-free foods; and guidance for specific foods. Responses were divided into paediatric and adult groups. Initially, there was majority consensus (≥86%) by paediatric dietitians (*n* = 29) for 14 of 16 statements; a further 2 structured discussions were required for 2 statements, with a final majority consensus of 72% (*n* = 26/36) and 64% (*n* = 16/25). In adult practice, 75% of dietitians agreed with all initial statements for adult patients and 40% advocated separate maternal-PKU guidelines. In Phase 2, 5 of 6 statements were agreed by ≥76% of respondents with one statement requiring a further round of discussion resulting in 2 agreed statements with a consensus of ≥71% by dietitians in both paediatric and adult practice. In Phase 3 one statement was added to elaborate further on an initial statement, and this received 94% acceptance by respondents. Statements were endorsed by the UK National Society for PKU.

**Conclusions:**

The BIMDG dietitians group have developed consensus dietetic statements that aim to harmonise dietary advice given to patients with PKU across the UK, but monitoring of statement adherence by health professionals and patients is required.

## Introduction

Phenylketonuria (PKU) is an inborn error of amino acid metabolism, due to deficiency or absence of the enzyme phenylalanine hydroxylase, leading to accumulation of blood and brain phenylalanine (Phe). Untreated, it will cause severe, irreversible neurological damage [1]. Strict dietary management is the only available treatment option in the UK. The aim is to correct abnormal biochemistry by decreasing the Phe load on the affected pathway and supplementation with Phe -free L-amino acids or low Phe glycomacropeptide protein substitutes [2]. The diet involves avoiding high protein foods (e.g. meat, fish, eggs, cheese, seeds, flour, bread and nuts), with strict control of moderate containing protein foods (e.g. cereals, potato, milk and some vegetables) to maintain blood Phe levels within target range [1, 3]. Several fruits and vegetables are low in Phe and are incorporated in the diet without limit [4]. From weaning when solids are first introduced [5] and later with the ever growing convenience food market, patients with PKU and their caregivers need simple, consistent, easy-to-understand rules for calculating protein intake. A national dietetic re-appraisal of some of the practical dietary advice given to UK patients and families with PKU was necessary because of: the recent publication of European PKU guidelines identifying different criteria for the allocation of fruit and vegetables in a low Phe diet [3]; introduction of new species of fruits and vegetables; new European protein labelling legislation [6]; contradictory information available via social media; and increasing patient usage of manufactured foods.

The British Inherited Metabolic Diseases (BIMDG) Dietitians Group, using the Delphi method set out to agree a set of practical statements about the classifications of foods in a low Phe diet. The Delphi process is a tool used to gain a majority decision in a structured or systematic manor. It helps secure a collective view from a panel of experts about complex issues or problems where there is little or no definitive evidence [7, 8]. Experts respond to questionnaires over several rounds with a facilitator coordinating and summarising responses for feedback. Responses and feedback from each expert, guide the questions for further rounds. With each successive round the number of questions declines as the group moves toward consensus.

## Aims

To develop consensus statements about the practical allocation of foods and interpretation of protein food labelling in a low Phe diet for the management of PKU in the UK using the Delphi method to generate consensus.

## Methods

In November 2015, BIMDG dietitians convened to discuss the allocation of foods and interpretation of food labeling with the aim of developing consensus statements for PKU. A multiple-choice questionnaire, comprising 14 questions about dietary advice to UK patients or caregivers regarding the allocation of different foods (allowed without measurement, not allowed, or allowed as part of Phe exchanges) and interpretation of protein from food labels, was distributed to 70 BIMDG dietitians from 30 centres treating individuals with PKU. The results of this questionnaire identified 7 specific areas where dietetic practice was variable, leading to contradictory advice being issued to patients and caregivers. These 7 areas are identified in Table 1.

**Table 1:** Variation in dietary advice given to patients by BIMDG dietitians

**Areas of variation in dietary advice given to patients by BIMDG dietitians:**	
Use of different terminology to describe low protein foods that could be incorporated into the diet without measurement.	
Inconsistent advice to parents about the calculation of 1 g protein exchanges (equivalent to 50 mg phenylalanine) from protein labelling analysis of individual food portions i.e. dietitians were either rounding protein values > or < 1 g to the nearest 0.5 exchange.	
Use of different upper protein ‘cut off’ points for foods that could be given in the diet without measurement (exchange-free). There was inconsistent allocation of the following foods: herbs and spices; fats/oils; soya sauce; gravy; cooking sauces; vegetable crisps; sweets; and processed vegetables/vegetable sauces with protein containing ingredients (e.g. milk/wheat).	
Inconsistent allocation of manufactured foods that contained low protein (exchange-free) ingredients.	
Inconsistent allocation of special low protein foods that contained low protein (exchange-free) ingredients.	
Inconsistent allocation of fruits and vegetables (containing phenylalanine from 50 to 100 mg/100 g weight) in the diet. Some dietitians were allowing certain fruits and vegetables in the diet without measurement; others were permitting in restricted amounts only.	
Inconsistent interpretation of manufactured food labelling: e.g. some foods are labelled as containing 0 g protein even though some of the ingredients are protein sources. This includes foods containing protein ≤0.5 g/100 g (permissible by European law: Regulation (EU) No 1169/2011) [4] or protein < 1 g/100 g (permissible by USA law).	

Following a round table discussion of these results at a BIMDG dietitians meeting, 16 consensus statements for the practical dietary management of PKU were put forward and agreed in Phase 1. In Phase 2 a further 6 statements, and in Phase 3 one final statement was proposed. The Delphi method was then used to gain consensus from the BIMDG dietitians about each of the statements. The lead author acted as facilitator.

The 23 consensus statements on practical dietary management were circulated to all BIMDG dietitians by email. A written response of agreement or disagreement with each statement was then returned to the facilitator for analysis. After each round of circulation, a telephone conference was held with the BIMDG dietitians to provide collective feedback and the draft consensus statements were further adapted. Modified statements were then recirculated to the BIMDG dietitians with additional questions until a majority decision (> 60%) was obtained on each statement. Dietitians were given 8 weeks to respond in each Delphi round.

Approval of the final consensus statements was sought and received by the UK National Society for PKU (NSPKU).

## Results

### Phase 1: Round 1 of Delphi method

The 16 draft consensus statements on practical dietary management were prepared with accompanying notes explaining their rationale. They were then distributed for approval or non-approval of each statement to all BIMDG dietitians (73 dietitians: 43 paediatric practice, 20 adult practice and 10 caring for both adult and paediatric patients; from 30 inherited metabolic disorder [IMD] centres).

Replies were received from 40 dietitians (55% of BIMDG dietitians, from 23 centres, 77%) and results were distributed to all dietitians within 5 months of initial circulation. After the first round, there was majority consensus by paediatric dietitians (≥86%; *n* = 29) and dietitians working in adult practice (≥65%; *n* = 17) for all 16 statements. At a BIMDG dietitians group teleconference, minor modifications were made to a small number of the consensus statements and 14 of the 16 statements were formerly agreed by paediatric dietitians (Tables 2 and 3).

**Table 2:** Summary of Consensus Statements

**PHASE 1**						
Consensus statements	Delphi Process – **Round 1** % agreement (n)		Delphi process – **Round 2** % agreement (n)		Delphi process – **Round 3** % agreement (n)	
	Paediatric Dietitians *n* = 29 18 centres	Dietitians in adult practice *n* = 17 11 centres	Paediatric Dietitians *n* = 36 18 centres	Dietitians in adult practice *n* = 19 12 centres	Paediatric Dietitians *n* = 25 10 centres	Dietitians in adult practice *n* = 20 13 centres
In PKU, low protein, free, unmeasured or non-exchange foods are referred to as ‘exchange-free’ foods.	93 (27)	100 (17)	Agreed		Agreed	Agreed
**Foods are ‘exchange-free’ if protein content is ≤ 0.5 g/100 g of food:**						
• e.g. sweets	90 (26)	88 (15)	Agreed		Agreed	Agreed
• e.g. gravy	97 (28)	76 (13)	Agreed		Agreed	Agreed
**Exceptions that remain ‘exchange-free’ if > 0.5 g per 100 g or per 100 ml of food:**						
• all herbs, spices and seasonings (irrespective of the protein content on the food ingredient label, due to the small quantities consumed);	100 (29)	100 (29)	Agreed		Agreed	Agreed
• fats (oils, butter, margarine) (as only small amounts are used)	100 (29)	88 (15)	Agreed		Agreed	Agreed
• ‘wet cooking sauce’ if the protein content is ≤1.0 g/100 g	100 (29)	65 (11)	Agreed		Agreed	Agreed
**Soya sauce:**						
ROUND 1: soya sauce that contains Phe ≤ 1.0 g/100 ml (based on the quantities commonly consumed)	97 (28)	88 (15)	N/A		N/A	N/A
**ROUND 2:**						
• Option 1: Any soya sauce that contains protein ≤1.0 g/100 ml is considered ‘exchange-free’	N/A	N/A	25 (9)*		N/A	N/A
• Option 2: Any soya sauce that contains protein ≤1.5 g/100 ml is considered ‘exchange-free’	N/A	N/A	67 (24)*		Agreed	Agreed
• Option 3: 2 tablespoons per day of any soya sauce is ‘exchange-free’ (existing guideline)	N/A	N/A	14 (5)*		N/A	N/A
**For ‘wet cooking sauces’ with a protein content > 1.0 g /100 g:**						
• If they contain exchange ingredients (e.g. cream, egg, coconut) they are counted as an ‘**exchange’** food;	100 (29)	65 (11)	Agreed		Agreed	Agreed
• If they contain ‘exchange free’ ingredients only (e.g. fruit/vegetables) they are considered an ‘exchange-free’ food.	100 (29)	65 (11)	Agreed		Agreed	Agreed
***Allocation of fruits and vegetables (except fresh/frozen potatoes)***						
ROUND 1:						
• Fruits & vegetables (except potatoes) containing a Phe content ≤75 mg/100 g weight will be consisdered ‘exchange-free’ foods.	100 (29)	100 (17)	Agreed		Agreed	Agreed
• Fruits & vegetables with a Phe content of ≥100 mg/100 g, use the actual Phe content to calculate **exchange** amounts.	100 (29)	100 (17)	Agreed		Agreed	Agreed
• Phe content 76-99 mg/100 g weight of fruit & vegetable: allow 1 portion/day ‘exchange-free’	86 (25)	82 (14)	N/A		N/A	N/A
ROUND 2/3:						
• Option 1: Fruit & vegetables containing a Phe content 76–99 mg/100 g weight of fruit & vegetable, count as **‘exchange’** foods	N/A	N/A	35 (13)*		64 (16)	Agreed
• Option 2: Allow 1 exchange portion ‘exchange-free’ per day with additional portions to be counted as an **exchange**	N/A	N/A	42 (15)*		24 (6)	
• Option 3: Only 1 portion of these fruits/ vegetables are allowed ‘exchange-free’ per day (existing guideline)	N/A	N/A	33 (12)*			
Additional statement: Fruits & vegetables containing a Phe content of 76–99 mg/100 g, use a standard **‘exchange’** amount of 60 g weight to provide approximately 50 mg/Phe	N/A	N/A	97 (35)		Agreed	Agreed
• ***Fresh/frozen potatoes*** are counted as **‘exchange’** foods. If the Phe analysis is available, this will be used to determine exchange amount; if Phe analysis is unavailable (e.g. potato waffles, hash browns), the protein content will be used to calculate exchange amounts.	97 (28)	94 (16)	Agreed		Agreed	Agreed
• ***Manufactured fruit/vegetable*** products containing only fruits or vegetables that are designated as ‘exchange-free’ in their fresh form, should still be considered ‘exchange-free’ foods. However, if they contain added ingredients that are protein containing (e.g. milk or wheat), they are counted as **‘exchange’** foods with their protein content used to determine the amount of food allocated for one Phe exchange.	100 (29)	88 (15)	Agreed		Agreed	Agreed
• ***Vegetable crisps*** containing exchange free vegetables are counted as **‘exchange’** foods due to the concentration of protein associated with cooking methods; their protein content should be used to determine the exchange amount.	88 (21)	88 (15)	Agreed		Agreed	Agreed
**Low protein special foods:**						
• Low protein special products (e.g. bread, flour) should be allowed without measurement if all ingredients are ‘exchange-free’, irrespective of the phenylalanine analysis per 100 g on the label.	93 (27)	76 (13)	Agreed		Agreed	Agreed
***alculating food protein exchanges from protein analysis on the food label:***						
• Food **‘exchange’** amounts for food portions should be rounded up or down based on the ‘rule of maths’ (Table 3 for guidance).	97 (28)	94 (16)	Agreed		Agreed	Agreed
• Patients/caregivers are advised to read ingredient lists as well as the protein content on food labels. The following was concluded as guidance:	100 (29)	88 (15)	Agreed		Agreed	Agreed
- If ingredients contain protein but the protein label content appears low, establish a more accurate protein analysis before consuming.	100 (29)	88 (15)	Agreed		Agreed	Agreed
- If ingredients are ‘clearly’ exchange-free but the protein label content is not available then it is suitable to give as an exchange-free food.	100 (29)	100 (17)	Agreed		Agreed	Agreed
- If there is no protein content on the product label, but it contains **exchange** ingredients, an accurate protein analysis should be obtained before consuming.	100 (29)	100 (29)	Agreed		Agreed	Agreed
- If a label states its protein content is 0 g but ≥1 ingredient contains a protein source (e.g. gelatine), avoid until an accurate protein analysis is known.	97 (28)	65 (11)	Agreed		Agreed	Agreed
Agree with all consensus statements (as above)	N/A	N/A	N/A	N/A	N/A	75 (15)
Separate maternal PKU statements preferred	N/A	N/A	N/A	N/A	N/A	40 (8)
PHASE 2						
Consensus statements	Delphi Process – Round 1 % agreement (n)		Delphi process – Round 2 % agreement (n)		Final Decision	
	Paediatric Dietitians *n* = 19 13 centres	Dietitians in adult practice *n* = 9 8 centres	Paediatric Dietitians *n = 19* 11 centres	Dietitians in adult practice *n* = 14 8 centres		
**Low protein milk**						
**ROUND 1:**						
• Option 1: A daily volume of 250 ml is exchange free if it provides ≤0.5 g protein (25 mg Phe)/daily	16 (3)	56 (5)	N/A	N/A		
• Option 2: A daily volume of 500 ml/daily volume is exchange free if it provides ≤0.5 g protein (25 mg Phe) /daily	42 (8)	11 (1)	N/A	N/A		
• Option 3: A daily volume of 1000 ml/daily volume is exchange free if it provides ≤0.5 g protein (25 mg Phe) /daily	21 (4)	22 (2)	N/A	N/A		
• Option 4: A daily volume of 1000 ml/daily volume is exchange free if it provides ≤0.5 g protein (25 mg Phe) /daily	11 (2)	11 (1)	N/A	N/A		
• Unsure	11 (2)	–	N/A	N/A		
**ROUND 2:**						
• Any plant milk (e.g. coconut, rice or almond) that provides a total protein intake of > 0.5 g over 24 h when consumed should be counted as an exchange food. If the total protein intake provides ≤0.5 g over 24 h, it should be considered exchange-free.	N/A	N/A	84 (16)	79 (11)	Agreed	
• Any low protein special milk (e.g. Sno Pro, Taranis, Prozero) that provides a total phenylalanine intake of > 25 mg (half an exchange) over 24 h when consumed should be counted as an exchange food. If the total phenylalanine intake provides ≤25 mg over 24 h, it should be considered exchange-free.	N/A	N/A	79 (15)	71 (10)	Agreed	
***Soups*** that contain exchange-free ingredients are exchange-free.	79 (15)	78 (7)	N/A	N/A	Agreed	
Any ***coconut yoghurt/dessert*** with a protein content ≤0.5 g/100 g is exchange-free.	79 (15)	56 (5)	N/A	N/A	Agreed	
Any ***dried coconut product*** with a protein content > 0.5 g/100 g should be counted as an **exchange** food.	95 (18)	89 (8)	N/A	N/A	Agreed	
Any food containing ≤0.5 g/100 g protein (but contains **gelatine**) can be allocated as exchange-free as it is likely to contain ≤10 mg phenylalanine from this source.	95 (18)	89 (8)	N/A	N/A	Agreed	
The weight rather than the volume of ***ice-cream*** should be used to calculate the protein exchange amount.	100 (19)	100 (9)	N/A	N/A	Agreed	
PHASE 3						
Consensus statements	Delphi Process – Round 1 % agreement (n)				Final Decision	
	*n = 17* 13 centres					
**Low protein special foods**						
If the special low protein food contains exchange ingredients but contains up to 25 mg Phe/100 g, it is exchange-free. If the special low protein food contains exchange ingredients but contains ≥26 mg Phe/100 g, it is an exchange food.	94 (16)				Agreed	

NB: Dietitians covering both paediatrics & adults – responses are included in both groups. *some dietitians were undecided and chose 2 options

**Table 3:** Calculating food protein exchanges from protein analysis on the food label

Protein content per item when calculated from label	Calculated exchange
0 g protein per food portion	Exchange free
0.1 g protein per food portion	Exchange free (if total volume consumed is ≤0.5 g protein)
0.2 g protein per food portion	Exchange free (if total volume consumed is ≤0.5 g protein)
0.3 g protein per food portion	Exchange free (if total volume consumed ≤0.5 g protein). Suggest 1 portion is exchange-free, 2 portions is ½ exchange^a^
0.4 to 0.7 g protein per food portion	½ exchange protein
0.8 to 1.2 g protein per food portion	1 exchange protein
1.3 to 1.7 g per food portion	1.5 exchange protein
1.8 to 2.2 g per food portion	2 exchange protein
2.3 to 2.7 g per food portion	2.5 exchange protein
2.8 to 3.2 per food portion	3 exchange protein

^a^This may apply to ice cream lollies, gluten-free cakes, very small packets crisps, sweets

However, two of the statements generated considerable debate, so it was decided to further explore individual opinions on these 2 statements following additional investigation into the Phe content of the products. In addition, there was some disparity among dietitians working in adult practice in relation to maternal patients with PKU and Phe consumption from fruits and vegetables permitted without measurement. Consequently, it was decided that for round 2 of the Delphi process, paediatric dietitians and dietitians working in adult practice would separate to examine issues independently.

### Phase 1: Round 2 of Delphi method

The remaining 2 ‘non-agreed’ draft consensus statements (statement 1: the upper protein content of soya sauces allowed as an exchange-free food; and statement 2: the allocation of fruits and vegetables containing Phe content from 76 to 99 mg per 100 g weight) were modified following examination of barriers to acceptance (Table 2). An in-depth investigation was conducted about the protein content of all commercial soya sauces available to purchase as well as issuing analysis about the Phe content of fruits and vegetables. These 2 consensus statements were then recirculated to the BIMDG dietitians; with 3 possible options for each statement.

Soya sauce was considered differently to other table top sauces. Its protein content is highly variable from < 0.5 g per 100 ml up to 15 g per 100 ml. Soya sauce option 1: *protein ≤ 1 g per 100 ml is exchange-free,* was consistent with the statement for wet cooking sauces but allowed very little brand choice; option 2: *protein ≤ 1.5 g per 100 ml is exchange-free*, allowed more choice but was inconsistent with statements for other foods; and option 3: *up to 2 tablespoons per day of any soya sauce,* allowed a wide choice of brands but could lead to a higher protein intake.

For fruit and vegetables with a Phe content between 76 to 99 mg per 100 g: option 1: *count as part of the 50 mg Phe exchange system*, considered that their uncontrolled consumption may increase dietary Phe intake considerably, but may be difficult to enforce in patients already established on dietary management who do not currently restrict their intake. Option 2: *permit only one serving daily of any fruits and vegetables in this category, but calculate Phe intake from any additional servings of fruits and vegetables from this category,* overcame some of the issues associated with option 1, but was complex for both dietitians and patients or caregivers. Option 3: *permit 1 portion daily of any one of the fruits and vegetable in this category*, was the existing guidance and whilst aiming to control overall intake, it did not consider increased opportunities for consumption of some of the more novel forms of these fruits and vegetables e.g. vegetable rice, vegetable pasta. An additional question was asked in this round (Table 2) about the acceptance of a standard exchange weight i.e. 60 g for fruits and vegetables with a Phe content between 76 and 100 mg per 100 g.

For BIMDG paediatric dietitians, option 2 for the soya sauce consensus statement received a majority response (67% of respondents; *n* = 24/36) and was agreed; but opinion was divided between the 3 options for the final consensus statement on fruits and vegetables containing Phe content from 76 to 99 mg per 100 g weight. However, there was consensus on the need for a standard exchange weight for these fruits and vegetables (Table 4). The dietitians working in adult practice continued to debate both questions but did not reach consensus at this round.

**Table 4:** New exchanges (previously exchange free)

Fruit and vegetables containing Phe ≥ 76 mg/100 g	Amount for 1 exchange
Figs	60 g
Asparagus	60 g
Beansprouts	60 g
Broccoli	60 g
Brussel sprouts	60 g
Cauliflower	60 g
Yam	60 g
Sugar snap peas	60 g
Mange tout	60 g
Whole hearts of palm	60 g

### Phase 1: Round 3 of Delphi method

The remaining ‘non-agreed’ consensus statement concerning the allocation of fruits and vegetables was reissued with the 2-options scoring highest in the previous round. Option 1 statement was finally agreed by 64% (*n* = 25) of paediatric dietitians and endorsed at the dietitians BIMDG teleconference in February 2017.

In the final Delphi round, 75 % of dietitians working in adult practice (*n* = 15) agreed with the 16 consensus statements for adult patients following diet, concluding that consistency in dietary care in the transition from paediatric to adult services was important. However, 40% (*n* = 8) of dietitians working in adult practice proposed that separate maternal PKU consensus statements were required.

### NSPKU endorsement

The initial consensus statements were endorsed by the UK National Society for PKU (NSPKU) in April 2017.

### Phase 2: Round 1of Delphi method

Additional food items not covered by the initial statements were identified in the 6 months following completion of Phase 1, so 6 new statements on: low protein milks, soups, coconut desserts, coconut products, ice-cream and gelatine containing products, were distributed with accompanying notes. Replies were received from 25 dietitians (from 18 centres) and results were discussed at a BIMDG dietitians group teleconference in January 2018. There was majority consensus by dietitians (≥76%) for all but the low protein milks statement which it was agreed needed redefining (Table 2).

### Phase 2: Round 2 of Delphi method

Two amended statements on plant milks and low protein milk replacements were sent out in February 2018 and results discussed and agreed at a teleconference in May 2018. There were 30 replies and 77% (*n* = 23) agreed with the plant milks statement and 80% (n = 24) with the low protein milk replacements statement (Table 2). Other low protein special foods were also discussed in detail and it was agreed that the initial statement in Phase 1 required elaboration due to significant protein containing ingredients in some products.

### Phase 3: Round 1 of Delphi method

A statement regarding low protein special foods was distributed along with information on their content in May 2018. Results were conclusive and this along with Phase 2 statements were agreed at a BIMDG dietitians meeting in June 2018. A summary of all agreed guidelines was issued in July 2018 and endorsed by the NSPKU (Table 5).

**Table 5:** Summary of Final Consensus Statements for PKU

1. Any food given without measurement is referred to as an exchange-free food.	
2. Foods are ‘exchange-free’ if they contain protein ≤0.5 g/100 g. e.g. sweets, coconut products, foods containing gelatine. Exceptions: • herbs, spices, seasonings, fats (oil, butter, margarine) – as the quantity used is very small. • Any soya sauce containing protein ≤1.5 g/100 ml is exchange-free. • Any ‘wet cooking sauce’ containing protein ≤1.0 g/100 g is exchange-free. If it contains > 1 g protein/100 g and contains exchange ingredients (e.g. cream, egg, coconut) it should be counted as an ***exchange food****. If it contains > 1 g protein/100 g and contains ‘exchange free’ ingredients only (*e.g. vegetables such as tomatoes) it is an exchange-free food.	
3. ‘European PKU guideline 2017’ [3] is used for fruit and vegetable allocation: • Phe content ≤75 mg/100 g weight of fruit and vegetables: exchange-free. • Phe content ≥76 mg/100 g weight of fruit and vegetables: count as **exchange foods**. Exception: Potatoes – use Phe analysis to determine exchange amounts. If potato products contain additional exchange ingredients (e.g. wheat flour, or milk), use protein analysis on the packet to determine its exchange amount.	
4. A standard exchange amount of 60 g for any fruit/vegetables containing Phe between 76 and 99 mg/100 g will be used. For any fruit/vegetables containing Phe ≥100 mg/100 g (e.g. peas, sweetcorn), the actual Phe content will be used to calculate exchange amounts.	
5. If any frozen/canned product is designated ‘exchange-free’ in their fresh form, they are considered exchange-free foods e.g. carrots, mushrooms, tomatoes. Exception: Vegetable crisps - although derived from exchange-free food, are concentrated in protein due to cooking methods so should be counted as **exchange foods**. Use protein content per 100 g to determine the amount that can be given for one exchange.	
6. Low protein special products (e.g. bread, flour) are exchange-free if all ingredients are exchange-free. If they contain exchange ingredients but contains ≤25 mg Phe/100 g, they are exchange-free. If they contain exchange ingredients but contains ≥26 mg Phe/100 g, they are an **exchange food**.	
7. Any low protein special milk that provides a total Phe intake of > 25 mg (1/2 exchange) over 24 h in the volumes consumed, should be counted as an **exchange food**. If the total Phe intake provides ≤25 mg over 24 h, it should be considered exchange-free.	
8. Any plant milk (e.g. coconut, rice, almond) that provides a total protein intake of > 0.5 g over 24 h in the volumes consumed, should be counted as an **exchange food**. If the total protein intake provides ≤0.5 g/100 g over 24 h, it should be considered exchange-free.	
9. Soups that contain exchange-free ingredients are exchange-free. If soups contain exchange ingredients and their protein content is > 0.5 g/100 g, then they should be counted as an **exchange food**.	
10. Weight rather than the volume of ice-cream should be used to calculate the protein exchange amount.	
11. Food ‘exchange’ amounts for food portions should be rounded up or down based on the ‘rule of maths’. See Table 3	
12. All patients/caregivers are advised to read ingredient lists as well the protein content/100 g on food labels. If ingredients are protein containing (but the protein analysis appears very low), it is important to establish more accurate protein analysis before consuming. If ingredients are ‘clearly’ exchange-free, then it is appropriate to give as an exchange-free food even if the protein analysis is unavailable on the label. If there is no nutritional analysis on the product but it contains ingredients that are **exchange foods**, then further food protein analysis must be obtained before consuming. If a label states protein content is 0 g but one or more ingredients is a protein source (e.g. gelatine), then an accurate food protein analysis must be obtained before consuming.	

## Discussion

National consensus statements on the practical interpretation of dietary management in PKU are central to ensuring consistent advice is given to all families and patients with PKU. We have systematic agreement on many basic dietary rules and definitions which have received united support from BIMDG dietitians. Using the Delphi method allowed all BIMDG dietitians to have the opportunity to give their opinion and influence several areas of dietary management in PKU where scientific evidence was unavailable to dictate practice. For most of the statements, clear consensus was reached in the first round of Delphi discussions, leaving only 4 controversial statements requiring more extensive discussion and negotiation. The process we have undertaken should lead to harmonization and consistency of dietetic practice in PKU with less confusion for professionals, patients and their families. Adoption of these consensus statements by the national patient’s society, NSPKU, will enable uniform written dietary information to be available for all patients. Further adaptation of these statements is necessary for maternal PKU, where dietary management practices are particularly rigorous. This exercise will be taken forward by the BIMDG dietitians working in adult practice.

The advantage of the Delphi method is that it involves the collective knowledge of a group of experts which is likely to be better than that of each individual; comparing, contrasting, challenging and complementing each other [8]. The process is directed, impartial, helps engender group ownership, encourages consensus among individuals with diverse views and is an alternative to conventional meetings where strong personalities, status and group pressures can influence individual responses [7]. It was clear that every opinion was valued and would be important in influencing the final decisions. Because this method is structured and focused, it can avoid much of the counterproductive digressions identifiable in face-to-face group discussions [7]. Due to the combined contribution of all members, varying ideas and viewpoints were generated and these further directed later responses.

The Delphi Method did have some limitations. Being a qualitative method, it is considered subjective. However, in our consensus statements, only best practice opinion was available to support any of the specific decisions reached; when new scientific evidence is available these statements will be challenged. Also, 70 dietitians participated, which could be considered a limitation. However, the number of experts chosen was designed to be inclusive of all centres and dietitians in the UK who were members of the BIMDG dietitians group. Another drawback of the process was the tendency for participants to maintain the status quo rather than voting for change. For example, for two of the consensus statements, despite good initial agreement in round 1, further discussion identified the impracticality of the statements which prompted the generation of 3 possible options for round 2. A further limitation was the lack of response from some members. Unfortunately, this was unavoidable due to maternity leave, job changes and retirements occurring during the 18-month period of the project. Some dietetic non-responders were new to the IMD specialty or were from centres with fewer patients and or less clinical experience and may have been reluctant to respond due to uncertainty or a lack of definitive opinion. Despite some non-responding dietitians, most UK IMD centres were represented in each round.

## Conclusion

In conclusion, this process of agreement between BIMDG dietitians across the UK will enable the introduction of consistent, easy-to-understand rules for calculating protein intake for professionals and patients with PKU. Both dietitians working in paediatric and adult services have endorsed these consensus statements which should contribute to a smooth transition between services, maintaining uniformity of information across all ages. Modification of the consensus statements may be warranted for maternal PKU patients requiring more rigorous dietary restriction. It will be important to perform an evaluation of the interpretation of these statements by dietitians and patients in clinical practice. Overall these consensus statements contribute to harmonising dietary advice offered to British PKU patients. Longitudinal monitoring of their application, acceptance and adherence by health professionals and patients or caregivers is essential.

## Abbreviations

BIMDG: British Inherited Metabolic Diseases Group
IMD: Inherited Metabolic Diseases
NSPKU: National Society for Phenylketonuria
Phe: Phenylalanine
PKU: Phenylketonuria
